# RNA methyltransferase NSUN2 promotes stress-induced HUVEC senescence

**DOI:** 10.18632/oncotarget.8087

**Published:** 2016-03-15

**Authors:** Xiaoyu Cai, Yuanyuan Hu, Hao Tang, Han Hu, Lijun Pang, Junyue Xing, Zhenyun Liu, Yuhong Luo, Bin Jiang, Te Liu, Myriam Gorospe, Chuan Chen, Wengong Wang

**Affiliations:** ^1^ Department of Biochemistry and Molecular Biology, Peking University Health Science Center, Beijing, P. R. China; ^2^ Department of Physiology and Pathophysiology, Peking University Health Science Center, Beijing, P.R. China; ^3^ Laboratory of Genetics, National Institute on Aging, National Institutes of Health, Baltimore, MD, USA; ^4^ Shanghai Geriatric Institute of Chinese Medicine, Shanghai, China

**Keywords:** NSUN2, SHC mRNA methylation, translational regulation, HUVEC, premature senescence, Gerotarget

## Abstract

The tRNA methyltransferase NSUN2 delays replicative senescence by regulating the translation of *CDK1* and *CDKN1B* mRNAs. However, whether NSUN2 influences premature cellular senescence remains untested. Here we show that NSUN2 methylates *SHC* mRNA *in vitro* and in cells, thereby enhancing the translation of the three SHC proteins, p66SHC, p52SHC, and p46SHC. Our results further show that the elevation of SHC expression by NSUN2-mediated mRNA methylation increased the levels of ROS, activated p38MAPK, thereby accelerating oxidative stress- and high-glucose-induced senescence of human vascular endothelial cells (HUVEC). Our findings highlight the critical impact of NSUN2-mediated mRNA methylation in promoting premature senescence.

## INTRODUCTION

The SHC family of proteins consists of three isoforms, p66SHC, p52SHC, and p46SHC, that arise through alternative initiation of translation [1]. Initially, all three SHC isoforms were found to form a complex with Grb2 and thus functioned as “adaptor” proteins in the Ras signaling pathway. However, unlike p52SHC and p46SHC, p66SHC contains a unique N-terminal collagen homology domain (CH2), which confers to p66SHC functions besides that of activator of the Ras signal pathway [2]. For example, phosphorylation of Ser36, which is located at the CH2 domain, triggers ROS (reactive oxygen species) production, a critical function of p66SHC in determining the longevity of mammals [3, 4]. In addition, p66SHC may also play important roles in human cancer and aging, since elevation of p66SHC protein has been observed in primary human prostate tumors [5], in replicative senescence [6, 7], in middle-aged mice [8], as well as in cells exposed to oxidative stress and to short-wavelength ultraviolet irradiation (UVC) [9, 10]. p66SHC levels are regulated by enhanced protein turnover mediated by the tumor suppressor TP53 (p53) and by suppression of transcription following DNMT3b-mediated methylation of the SHC promoter region [11, 12]. Recently, we showed that microRNA let-7 represses the translation of p66SHC in replicative senescence, the type of senescence that is triggered by the critical shortening of telomeres after a finite round of divisions [6]. However, whether SHC expression influences cell senescence triggered by other factors was not studied.

Methylation is a prevalent post-transcriptional modification of RNAs [13, 14] and influences the efficiency and accuracy of mRNA translation [15, 16], the half-life of RNAs [17, 18], and the biogenesis of small RNAs [17, 19, 20]. NSUN2 (NOP2/Sun domain family, member 2; MYC-induced SUN domain-containing protein, Misu) mediates MYC-induced cell proliferation [21]. In previous studies, we have reported that NSUN2 methylates the 3′UTRs of mRNAs encoding p16 (*CDKN2A*), TP53, E2F3, and ErbB2, thereby enhancing the expression of these proteins in cells exposed to oxidative stress [22, 23]. NSUN2-mediated mRNA methylation could also promote cell proliferation by elevating CDK1 translation [24]. More recently, we reported that NSUN2 could delay replicative senescence by methylating *CDK1* mRNA and *CDKN1B* (*p27^KIP1^*) mRNA [25]. However, whether or not NSUN2 influences premature cellular senescence, the senescence program triggered by acute exposure to stress conditions (such as oxidative stress, genotoxic damage, and high glucose levels) and the mechanisms underlying have not been reported.

In the present study, we provide evidence that NSUN2 methylates *SHC* mRNA at the 5′UTR (untranslated region), CR (coding region), and 3′UTR. Methylation by NSUN2 enhances the translation of *SHC* mRNA, thereby accelerating the senescence of HUVEC (human umbilical vein endothelial cells) in response to oxidative stress and high glucose. NSUN2-mediated mRNA methylation and regulation of TP53 and p16 expression levels were also observed after oxidative stress- and high glucose-induced HUVEC senescence. In sum, by targeting different senescence-associated mRNAs, NSUN2 is able to exert opposite roles in premature senescence relative to replicative senescence.

## RESULTS

### NSUN2 positively regulates SHC expression levels

**Figure 1:**
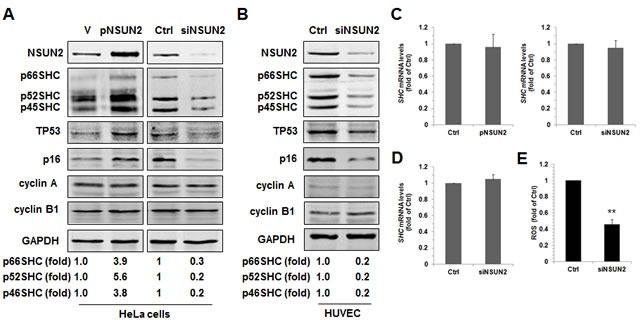
NSUN2 regulates SHC expression **A.** HeLa cells were transfected with a vector expressing NSUN2 or with a siRNA targeting NSUN2. Forty eight hours later, cell lysates were prepared and subjected to Western blot analysis to assess the levels of proteins NSUN2, p66SHC, p52SHC, p46SHC, TP53, p16, cyclin A, cyclin B1, and GAPDH. Data are representative from three independent experiments. **B.** HUVECs were transfected with a siRNA targeting NSUN2. Forty eight h after transfection, Western blot analysis was performed to assess the protein levels of NSUN2, p66SHC, p52SHC, p46SHC, TP53, p16, cyclin A, cyclin B1, and GAPDH. Data are representative from three independent experiments. **C.,D.** RNA samples described in panels A (C) and B (D) were subjected to RT-qPCR analysis to assess the levels of *SHC* mRNA. Data represent mean ± SD from 3 independent experiments. **E.** The cellular ROS level in cells described in Figure 1B were determined. Data represent mean ± SD from 3 independent experiments; significance was analyzed by using Students' *t* test (**, *p* < 0.01).

To test if SHC expression could be regulated by NSUN2-mediated mRNA methylation, we tested SHC protein levels in HeLa cells in which NSUN2 was overexpressed or silenced. As shown in Figure 1A, overexpression of NSUN2 increased the levels of SHC proteins p66SHC, p52SHC, and p46SHC (~3.9-, ~5.6-, and ~3.8-fold, respectively), while NSUN2 knockdown decreased the same (by ~70%, ~80%, and ~80%, respectively). In keeping with previous studies [22-23], TP53 and p16 protein levels increased in HeLa cells overexpressing NSUN2, but decreased in cells in which NSUN2 was silenced. As controls, neither overexpression nor knockdown of NSUN2 altered the levels of proteins cyclin A, cyclin B1, or GAPDH. These results suggest that NSUN2 may act as a positive regulator for the expression of SHC. We next asked if the regulation of SHC, TP53, and p16 by NSUN2 could be also observed in HUVECs. As shown, knockdown of NSUN2 in HUVECs greatly reduced the protein levels of SHC (~80% for p66SHC, p52SHC, and p46SHC), TP53, and p16, suggesting that NSUN2 also regulated the expression of SHC, TP53, and p16 in HUVECs (Figure 1B). Given that the expression of SHC could be regulated by TP53 at the level of protein turnover and NSUN2 can modulate TP53 translation [11, 23, 25], we tested if NSUN2 could also regulate the expression of SHC in a TP53-independent manner. As shown in Supplemental Figure S1, knockdown of NSUN2 in TP53-deficient human colorectal carcinoma HCT116 cells (HCT116 −/−) still reduced the levels of SHC proteins, suggesting that NSUN2 is able of regulating SHC expression in a TP53-independent manner. p52SHC and p46SHC were translated from the same transcript as p66SHC (*SHC* mRNA) by alterative initiation of translation [1]. The levels of *SHC* mRNA, which could potentially be used for synthesis of all SHC proteins, were not substantially altered by modulating NSUN2 abundance (Figure 1C and 1D), suggesting that NSUN2 does not affect SHC expression at the level of mRNA turnover and transcription and instead may affect SHC translation. In agreement with the findings that p66SHC is implicated in the production of intracellular ROS (reactive oxygen species) [3, 4], ROS levels were significantly decreased in HUVECs in which NSUN2 was silenced (by ~54%, *p* < 0.01) (Figure 1E).

### NSUN2 methylates *SHC* mRNA at multiple sites *in vitro* and in cells

**Figure 2:**
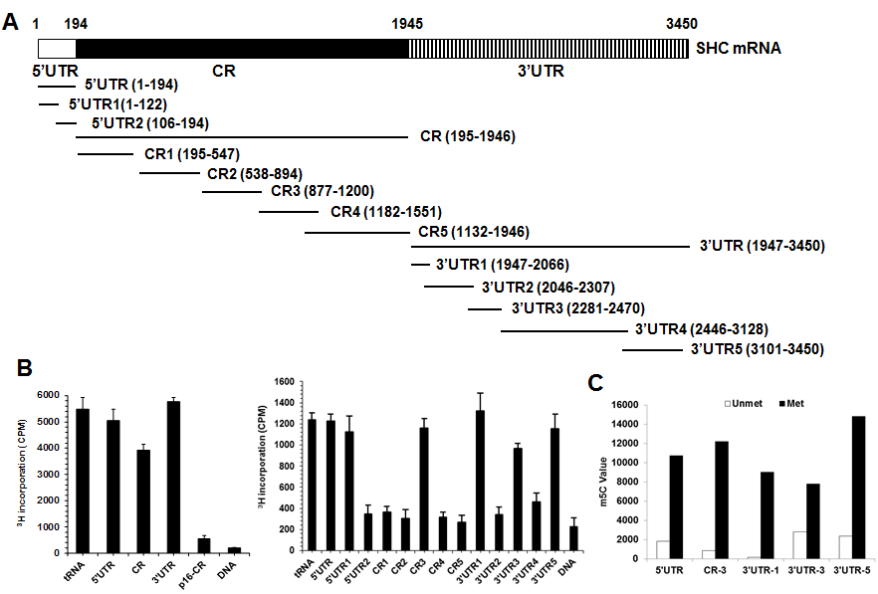
NSUN2 methylates *SHC* mRNA *in vitro* **A.** Schematic representation depicting the *p66SHC* mRNA fragments used for *in vitro* methylation assays. **B.** Incorporation of ^3^H-labeled SAM into *p66SHC* 5′UTR, CR, and 3′UTR fragments (left) as well as 5′UTR, 5′UTR1, 5′UTR2, CR1, CR2, CR3, CR4, CR5, 3′UTR1, 3′UTR2, 3′UTR3, 3′UTR4, and 3′UTR5 fragments (right). The incorporation of ^3^H-labeled SAM into p66SHC cDNA (DNA) and p16-CR (coding region) served as negative controls. The incorporation of ^3^H-labeled SAM into bacteria tRNA served as a positive control. **C.** *p66SHC* 5′UTR, CR3, 3′UTR1, 3′UTR2, and 3′UTR3 fragments were *in vitro* methylated by non-isotopic SAM (Met) or kept untreated (Unmet), whereupon these fragments were subjected to HPLC-MS analysis to determine the formation of m5C. Data present the peak value of m5C.

**Figure 3:**
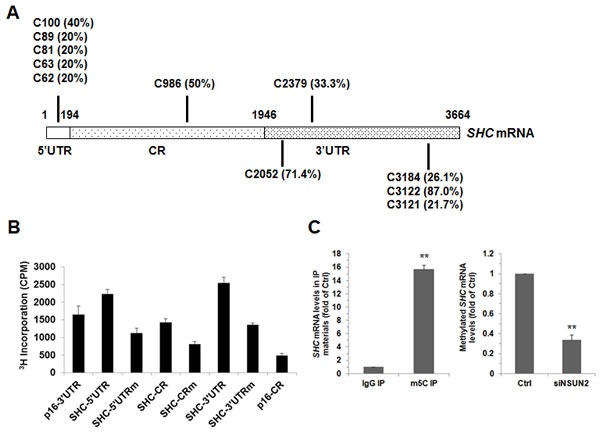
NSUN2 methylates *SHC* mRNA in cells **A.** *In vitro* methylated 5′UTR1, CR3, 3′UTR1, 3′UTR2, and 3′UTR5 fragments were subjected to bisulfate RNA sequencing analysis to identify the methylation sites, as described in the Materials and Methods section. The percentage of methylation of each identified site (more than 20%) was indicated. **B.** Incorporation of ^3^H-labeled SAM into the 5′UTR, CR, and 3′UTR and their variants with methylated sites (5′UTRm, CRm, and 3′UTRm). *P16* 3′UTR and CR fragments were served as a negative and a positive control, respectively. **C.** Left, RNA isolated from HUVECs was subjected to IP using anti-m5C or IgG antibodies. The presence of *SHC* mRNA in the IP materials was analyzed using RT-qPCR. Right, RNA isolated from cells described in Fig. 1B was subjected to IP assays by using anti-m5C antibody, the presence p66SHC mRNA in the IP materials was analyzed by RT-qPCR Data represent the means ± SD from 3 independent experiments; significance was analyzed by Student's *t* test (**, *p* < 0.01).

NSUN2 has been shown to regulate the expression levels of TP53, p16, p27, and CDK1 by methylating the mRNAs that encode these proteins [22-25]. To test if NSUN2 methylates *SHC* mRNA, the *SHC* mRNA fragments described in Figure 2A were used for i*n vitro* methylation assays (Materials and Methods). The *SHC* cDNA (DNA) and *p16* (*CDKN2A*)-CR (coding region of *p16* mRNA) were included as negative controls, while bacterial tRNA served as a positive control. As shown in Figure 2B, tRNA, 5′UTR, 5′UTR1, CR, CR3, 3′UTR, 3′UTR1, 3′UTR3, and 3′UTR5 were methylated, while *p16*-CR, *SHC* cDNA, 5′UTR2, CR1, CR2, CR4, CR5, 3′UTR2, and 3′UTR4 were not methylated. Accordingly, the methylation sites were located at the *SHC* 5′UTR1 (positions 1-122), CR3 (positions 877-1200), 3′UTR1 (positions 1947-2066), 3′UTR3 (positions 2281-2470), and 3′UTR5 (3101-3450). To determine the formation of m5C or m6A in the methylated fragments, *SHC* 5′UTR1, CR3, 3′UTR1, 3′UTR3, and 3′UTR5 were methylated *in vitro* by using nonisotopic S-Adenosyl methionine (SAM) and NSUN2 or kept unmethylated (same reaction but without adding NSUN2) and subjected to MS-HPLC analysis. As shown, m5C was detected in the methylated *SHC* 5′UTR1, CR3, 3′UTR1, 3′UTR3, and 3′UTR5 fragments (Figure 2C and Supplemental Figure S2). Identification of m6A from the methylated *SHC* 5′UTR1, CR3, 3′UTR1, 3′UTR3, and 3′UTR5 fragments did not yield any positive results (not shown). To further identify the methylation sites in the *SHC* mRNA, *in vitro* methylated 5′UTR1, CR3, 3′UTR1, 3′UTR3, and 3′UTR5 fragments were subjected to bisulfate sequencing analysis. As shown in Figure 3A, the 5′UTR contained 5 potential methylation sites (C62, 20%; C63, 20%; C81, 20%; C89, 20%, C100, 40%), the CR fragment contained one potential methylation site (C986, 50%), and the 3′UTR contained 5 potential methylation sites (C2052, 71.4%; C2379, 33.3%; C3121, 21.7%; C3122, 87.0%, C3184, 26.1%).

To confirm the methylation sites of *SHC* mRNA, a mutant of 5′UTR (*SHC* 5′UTRm) with all 5 methylation sites mutated C to G (C62G, C63G, C81G, C89G, and C100G), a mutant of CR (*SHC* CRm) with mutated C986G, and a mutant of 3′UTR (*SHC* 3′UTRm) with the 5 methylation sites mutated (C2152G, C2379G, C3121G, C3122G, and C3184G) were used for *in vitro* methylation assays. As shown in Figure 3B, compared with their wild-type forms, the methylation of *SHC* 5′UTRm, *SHC* CRm, and *SHC* 3′UTRm was greatly reduced, but did not disappear. These results confirm the methylation sites identified in Figure 3A, and suggest the existence of methylation sites other than those of the major methylation sites in Figure 3A.

To test whether NSUN2 methylates *SHC* mRNA in cells, RNA isolated from the HUVECs was immunoprecipitated using an anti-m5C antibody. The presence of *SHC* mRNA in the IP materials was analyzed using reverse transcription (RT) and real-time quantitative (q)PCR. As shown in Figure 3C (*left*), *SHC* nRNA was strongly enriched in anti-m5C IP compared with anti-IgG (negative control) IP. The level of methylated *SHC* mRNA decreased significantly in cells silenced with NSUN2 (by ~66%, *p* < 0.01). In sum, NSUN2 methylates *SHC* mRNA in HUVEC cells.

### Methylation by NSUN2 enhances the SHC translation

**Figure 4:**
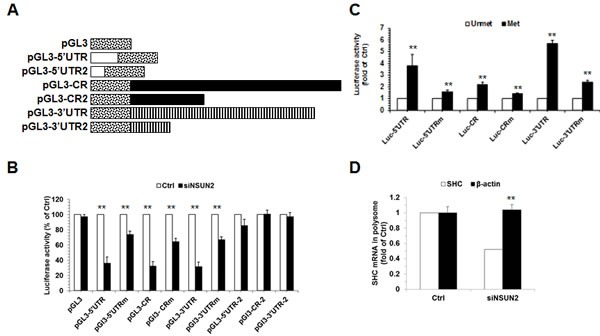
Methylation of *SHC* mRNA by NSUN2 enhances SHC translation **A.** Schematic representation depicting the pGL3-derived reporter vectors used for reporter gene assays. **B.** HeLa cells were transfected with each of the reporter vectors described in Figure 4A together with a pRL-CMV control reporter. Twenty-four hours later, cells were further transfected with with a siRNA targeting NSun2 and cultured for an additional 48 h. Firefly luciferase activity against Renilla luciferase activity was analyzed. Data represent the means ± SD from 3 independent experiments; significance was analyzed by Student's *t* test (**, *p* < 0.01). **C.** *In vitro* methylated (Met) or unmethylated (Unmet) luc-5′UTR, luc-5′UTRm, luc-CR, luc-CRm, luc-3′UTR, and luc-3′UTRm reporter transcripts were used for *in vitro* translation assays. Firefly luciferase activity was measured to reflect the translation efficiency. Data represent the means ± SD from 3 independent experiments; significance was analyzed by Student's *t* test (**, *p* < 0.01). **D.** Cells described in Figure 1B were used for isolating the polysomal fractions. RNA prepared from the fractions was subjected to RT-qPCR analysis to assess the presence of *SHC* mRNA and *β-Actin* (*ACTB*) mRNA in the polysomal fraction. Data represent the means ± SD from 3 independent experiments; significance was analyzed by Student's *t* test (**, *p* < 0.01).

Next, we asked if methylation by NSUN2 influences SHC expression levels. To this end, we first constructed a series of pGL3-derived reporters (Figure 4A, schematic) and tested the activity of these reporters in HeLa cells with silenced NSUN2. As shown in Figure 4B knockdown of NSUN2 reduced significantly the activity of these reporters (*p*<0.01). As controls, knockdown of NSUN2 could not alter significantly the activity of pGL3, pGL3-5′UTR2, pGL3-CR2, and pGL3-3′UTR2. Notably, the effect of NSUN2 knockdown in altering the luciferase activity from pGL3-5′UTRm, pGL3-CRm, and pGL3-3′UTRm was less effective, but not lost, in keeping with the results shown in Figure 3B. In sum, methylation by NSUN2 enhances SHC expression.

Because NSUN2 regulates the expression of p66SHC without influencing the levels of *SHC* mRNA (Figure 1A-1D), we further asked if methylation by NSUN2 regulated the translation of SHC. To this end, *in vitro*-transcribed reporter transcripts Luc-5′UTR, Luc-5′UTRm, Luc-CR, Luc-CRm, Luc-3′UTR, and Luc-3′UTRm (transcribed from pGL3-5′UTR, pGL3-5′UTRm, pGL3-CR, pGL3-CRm, pGL3-3′UTR, and pGL3-3′UTRm, respectively) were methylated *in vitro* by NSUN2 or kept unmethylated. These transcripts then were used to assay translation *in vitro* using rabbit reticulocyte assays and luciferase activities were measured as readout of the efficiency of translation. As shown in Figure 4C, methylation by NSUN2 increased the reporter activity of Luc-5′UTR (by ~3.8 fold, *p* < 0.01), Luc-CR (by ~2.2 fold, *p* < 0.01), and Luc-3′UTR (by ~5.7 fold, *p* < 0.01). In contrast, methylation by NSUN2 exhibited less pronounced effect on elevating the activity of Luc-5′UTRm (Luc-5′UTRm, ~1.6 fold, *p* < 0.01; Luc-CRm, ~1.4 fold, *p* < 0.01; Luc-3′UTRm, ~2.4 fold, *p* < 0.01). Moreover, fractionation of the polysomal component of the cytoplasm (Materials and Methods) followed by detection of *SHC* mRNA (as well as transcript *ACTB* mRNA, encoding the housekeeping protein β-Actin) revealed that NSUN2 is required for recruitment of *SHC* mRNA to polysomes (Figure 4D). Therefore, methylation of *SHC* mRNA by NSUN2 enhances the expression of SHC at the level of translation.

### NSUN2-mediated rise in SHC impacts on oxidative stress- and high glucose-induced HUVEC senescence

**Figure 5:**
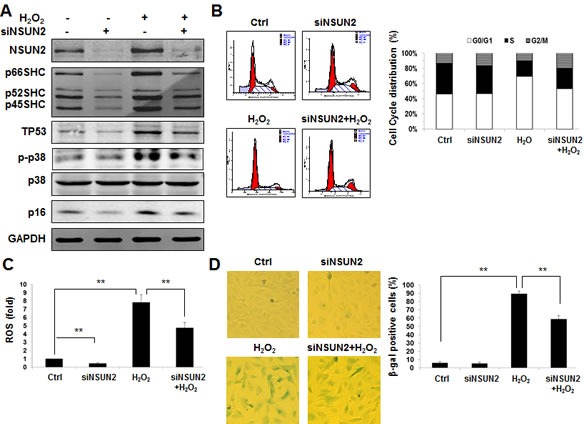
The NSUN2-SHC regulatory axis impacts on oxidative stress-induced cellular senescence **A.** HUVECs were transfected with an NSUN2 siRNA or a control siRNA. Twenty-four h later, cells were exposed to H_2_O_2_ (30 μM) and cultured for an additional 48 h. Cell lysates were prepared and subjected to Western blot analysis to assess the levels of p66SHC, p52SHC, p46SHC, p38, p-p38, p16, TP53 and GAPDH. **B.** Cells described in Figure 5A were subjected to FACS analysis. Data are representative from 3 independent experiments. **C.** Cellular ROS levels in cells described in Figure 5A were analyzed. Data shown are the mean ± SD from 3 independent experiments and statistical significance was analyzed by Student's *t* test (**, *p* < 0.01). **D.** Cells described in Fig. 5A were subjected to SA-β-gal analysis. Data represent the mean ± SD from 3 independent experiments and statistical significance was analyzed by Student's t test (**, *p* < 0.01).

In addition to SHC, NSUN2 could also regulate the expression of senescence-associated proteins TP53, p16/CDKN2A, p27/CDKN1B, CDK1, and CDC25C. NSUN2-mediated regulation of p27, CDK1, and CDC25C, but not NSUN2-mediated regulation of TP53 and p16 regulatory processes, has been shown to impact on the process of replicative senescence. Because the levels of NSUN2 and SHC are inversely correlated during replicative senescence [6, 25], the NSUN2-SHC regulatory axis may not be responsible for the elevated expression of p66SHC in replicative senescence. Moreover, knockdown of SHC in HUVECs diminished the effect of oxidative stress in repressing cell growth, elevating ROS levels, increasing the G1 compartment, and inducing premature senescence (Supplemental Figures. S3 and S4). Therefore, we asked if NSUN2-mediated regulation of SHC might impact on premature senescence of HUVECs. To this end, HUVECs with silenced NSUN2 were exposed to hydrogen peroxide (H_2_O_2_) or left untreated. Seventy-two hours later, the levels of protein NSUN2, SHC (p66SHC, p52SHC, and p46SHC), TP53, p16, p-p38 (phospho-p38MAPK), p38, and GAPDH were assessed by using Western blot analysis. As shown in Figure 5A, knockdown of NSUN2 in HUVECs reduced the levels of proteins SHC, TP53, and p16, while the levels of NSUN2, SHC, TP53, and p16 increased in response to oxidative stress. Importantly, knockdown of NSUN2 greatly diminished the effect of oxidative stress in elevating SHC, TP53, and p16 expression and in activating p38 MAPK (p-p38) (Figure 5A). As anticipated, knockdown of NSUN2 mitigated the effect of hydrogen peroxide in increasing the levels of ROS, rising the G1 compartment and inducing premature senescence (Figure 5B-5D). Taken together, our findings indicate that NSUN2-mediated regulation of SHC, TP53, and p16 may influence oxidative stress-induced premature senescence.

**Figure 6:**
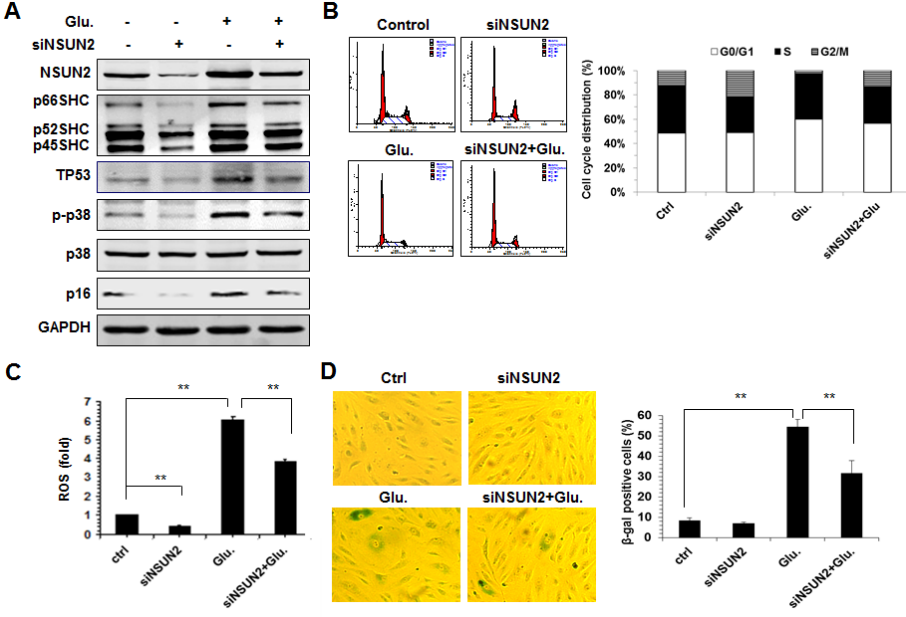
The NSUN2-mediated regulation of SHC impacts on high glucose-induced cellular senescence **A.** HUVECs were transfected with an NSUN2 siRNA or a control siRNA. Twenty-four h later, cells were exposed to glucose (33 mM) and cultured for an additional 48 h. Cell lysates were prepared and subjected to Western blot analysis to assess the protein levels of p66SHC, p52SHC, p46SHC, p38, p-p38, p16, TP53 and GAPDH. **B.** Cells described in Figure 6A were subjected to FACS analysis. Data are representative from 3 independent experiments. **C.** Cellular ROS levels in cells described in Figure 6A were analyzed. Data shown are the mean ± SD from 3 independent experiments and statistical significance was analyzed by Student's t test (**, *p* < 0.01). **D.** Cells described in Figure 6A were subjected to SA-β-gal analysis. Data represent the mean ± SD from 3 independent experiments and statistical significance was analyzed by Student's t test (**, *p* < 0.01).

Apart from oxidative stress, SHC proteins are also implicated in the process of premature senescence of HUVECs induced by high-glucose (Supplemental Figure S5). Thus, we further asked if NSUN2-regulated SHC levels influenced the high-glucose-induced premature senescence of HUVECs. To this end, HUVECs in which NSUN2 was silenced were treated with high glucose, and 72 h later, the levels of NSUN2, SHC (p66SHC, p52SHC, and p46SHC), TP53, p16, p-p38, p38, and GAPDH as well as the levels of cellular ROS, the cell cycle distribution, and the senescence-associated β-galactosidase [(SA)-β-gal] activity, were measured. As shown in Figure 6A-6D, exposure of HUVECs to high glucose increased the levels of SHC, TP53, p16, and p-p38, the levels of cellular ROS, the percentage of G1 cells, and SA-β-gal activity, while knockdown of NSUN2 mitigated all of these effects. In sum, NSUN2-regulated SHC, TP53, and p16 regulatory processes modulate the high-glucose-induced premature senescence.

## DISCUSSION

We have previously shown that NSUN2-mediated mRNA methylation delays the replicative senescence of human diploid fibroblasts (HDFs) by enhancing CDK1 translation and repressing p27 translation. In the present study, we provide evidence that NSUN2 enhances the translation of SHC proteins by methylating the 5′UTR, CR, and 3′UTR of *SHC* mRNA (Figures 1, 2, 3, 4). By elevating the expression of SHC, NSUN2 activates p38MAPK and increases the levels of cellular ROS, thereby regulating the premature senescence of HUVECs induced either by oxidative stress or by high glucose (Figures 5 and 6). Regulation of CDK1, p27, and CDC25C (but not TP53 or p16) by NSUN2 contributes to replicative senescence [25]. NSUN2 may not regulate SHC levels in replicative senescence, since senescent cells expressed low levels of NSUN2 but increased levels of SHC [6, 25]. By contrast, oxidative stress- and high-glucose-triggered cell senescence involved SHC-modulated TP53 and p16, but not NSUN2-modulated p27, CDK1, or CDC25C. Therefore, the function of NSUN2 in replicative senescence could be opposite to that in premature senescence, involving different regulatory processes.

SHC critically determines cellular life span and senescence [3, 4, 6]. Apart from NSUN2-mediated mRNA methylation, earlier studies demonstrated that methylation of the *SHC* promoter by DNMT3b silences *SHC* transcription [26]. However, our findings that exposure of HUVECs to oxidative stress and high glucose did not alter the levels of *SHC* mRNA or DNMT3b protein suggests that DNMT3 may not regulate p66SHC expression during premature senescence induced by oxidative stress and high glucose (Supplemental Figs S6A and S6B). On the other hand, let-7 has been shown to repress the translation of SHC in replicative senescence [6]. Interestingly, exposure of HUVECs to oxidative stress or high glucose decreases the levels of let-7 (Supplemental Figure S6C). Therefore, the let-7-mediated repression of SHC translation may also be involved in regulating SHC expression in oxidative stress- and high-glucose-induced premature senescence.

Although SHC suppresses the growth of HDFs [6] and HUVECs (Figs 5-6), SHC may promote the proliferation of human prostate cancer cells [5], in agreement with the view that cell senescence can contribute to tumorigenesis [27]. By elevating ROS production, SHC is also involved in vascular aging, high-glucose-induced endothelial dysfunction, hyperglycemia- and ROS-induced vascular dysfunction, and atherosclerosis induced by high-fat diet [28-32]. In line with these findings, the induction of NSUN2 and SHC in HUVECs in response to oxidative stress and high glucose (Figs 5-6 and Figs S4-S5) suggests that senescence achieved *via* the NSUN2-SHC regulatory axis may contribute to these pathological processes.

## MATERIALS AND METHODS

### Cell culture, FACS, transfection, and SA-β-gal staining

Human umbilical vein endothelial cells (HUVECs) were isolated from segments of human umbilical cord vein by collagenase digestion and cultured in medium 199 (Gibco) supplemented with 10% fetal bovine serum (FBS), as described [33]. HeLa cells were cultured in Dulbecco's modified Eagle's medium (Invitrogen) supplemented with 10% FBS, 100 units/ml penicillin, and 100 μg/ml streptomycin, at 37°C in 5% CO_2_. All plasmids were transfected using lipofectamine 2000 (Invitrogen) and cells were collected 48 to 72 h after transfection for further analysis. Fluorescence-activated cell sorting (FACS) analysis and SA-β-galactosidase staining was performed as described previously [6].

### Knockdown of NSUN2 and SHC

To silence NSUN2 and SHC, cells were transfected with siRNA (10 nmol/L) targeting NSUN2 (AGAUGUUAAGAUACUGUUGACCC), p66SHC (GCAGCCUAAGCAUUUGCUAdTdT), or with a control siRNA (UUGUUCGAACGUG UCACGUTT) using RNAiMAX (Invitrogen). Unless otherwise indicated, cells were collected for analysis 48 h after transfection. All knockdown interventions caused less than 1% cell death (by FACS analysis, data not shown).

### Measurement of the intracellular ROS

Intracellular ROS levels were measured using dichlorodihydrofluorescein diacetate (H2DCFDA, Invitrogen), as described [6]. Briefly, 1×10^4^ cells were plated into 96-well plates. Twenty-four hours later, cells were washed by HBSS (Hanks' Balanced Salt Solution) 3 times and incubated with the loading medium containing H2DCFDA (10 μM) at 37°C for 15 min. The density of fluorescence was measured at 488 nm excitation and 525 nm emission. The final results were corrected for variations in the cell numbers and expressed as fold of control.

### RNA isolation and RT-qPCR analysis

Total cellular RNA was prepared using the RNeasy Mini Kit (Qiagen). For reverse-transcription (RT) followed by real-time, quantitative (q)PCR or semiquantitative PCR analysis of the levels of *SHC* and *GAPDH* mRNAs, primers TGTCATCGCTGGAGGAAG and GAAGAAGGAGCACAGGGTAG for *SHC* mRNA and CGAGTCAACGGATTTGGTGGTAT and AGCCTTCTCCATGGTGAAGAC for *GAPDH* mRNA were used.

### Constructs and reporter gene assays

For construction of the pGL3-derived reporter vectors bearing the *SHC* 5′UTR and 5′UTRm (bearing mutations C62G, C63G, C81G, C89G, and C100G), the 5′UTR and 5′UTRm fragments were amplified by using primer pairs CCCAAGCTTGGGATGGGGCCTGAAACTGTCTG and CATGCCATGGCATGAGTTGAGGTGAAAGAGGGG and inserted into the HindIII and Nco I sites of the pGL3-promoter vector (Promega). For construction of the pGL3-derived reporter vectors bearing the *SHC* CR, CRm (bearing mutation C986G), 3′UTR, and 3′UTRm (bearing mutations C2052G, C2379G, C3121G, C3122G, and C3184G), the CR, CRm, 3′UTR, and 3′UTRm fragments were amplified by PCR by using primer pairs GCTCTAGAGCTGGATCTCCTGCCCCCCAA and GCTCTAGAGCTCACAGTTTCCGCTCCACAG (for CR and CRm) as well as GCTCTAGAGCTGTGGAGCGGAAACTGTGAT and GCTCTAGAGCTGCCTCTCAGTCTCGCGGT (for 3′UTR and 3′UTRm), and inserted into the XbaI site of the pGL3-promoter vector (Promega). The pcDNA 3.1 vector expressing NSUN2 was described previously [22].

For reporter gene assays, cell lysates were collected and the firefly and renilla luciferase activities were measured with a dual luciferase assay system (Promega) following the manufacturer's instructions. All firefly luciferase measurements were normalized to renilla luciferase measurements from the same sample.

### Preparation of polysomal fractions

A total of 20 million cells were incubated for 15 min with 100 μg/ml cycloheximide, and total lysates (500 μl) were layered onto a cushion of 30% sucrose in ice-cold buffer containing 20 mM HEPES (pH 7.4), 50 mM potassium acetate, 5 mM magnesium acetate, 1 mM dithiothreitol, 1 unit of RNasin per μl, 1 μg of leupeptin per ml, 1 μg of aprotinin per ml, and 0.5 mM phenylmethylsulfonyl fluoride. After centrifugation (Beckman SW40; 100,000 × *g* for 2 h, 4°C), RNA from the pellet (polysomal fraction) was isolated and used for RT-qPCR analysis.

### Transcript preparation

cDNA was used as a template for PCR amplification of the *SHC* mRNA fragments. All 5′ primers contained the T7 promoter sequence (CCAAGCTTCTAATACGACTCACTATAGGGAGA). To prepare templates for the *SHC* 5′UTR (positions 1-194), 5′UTR1 (positions 1-122), 5′UTR2 (positions 106-194), CR (positions 195-1946), CR1 (positions 195-547), CR2 (positions 538-894), CR3 (positions 877-1120), CR4 (positions 1182-1551), CR5 (positions 1532-1946), 3′UTR (positions 1947-3450), 3′UTR1 (positions 1947-2066), 3′UTR2 (positions 2046-2307), 3′UTR3 (positions 2281-2470), 3′UTR4 (positions 2446-3128), and 3′UTR5 (positions 3101-3450), the following primer pairs were used: (T7)ATGGGGCCTGAAACTGTCTG and AGTTGAGGTGAAAGAGGGG for 5′UTR, (T7)ATGGGGCCTGAAACTGTCTG and GTATCCCCAGGCCCTT for 5′UTR1, (T7)TAAGGGCCTGGGGATAC and AGTTGAGGTGAAAGAGGGG for 5′UTR2, (T7)TGGATCTCCTGCCCCCAA and TCACAGTTTCCGCTCCACAG for CR, (T7)TGGATCTCCTGCCCCCAA and CCGCCTCCACTCAGCTTG for CR1, (T7)GTGGAGGCGGCGGGCGCAG and AGAGTGATTGGCATTCCAG for CR2, (T7)CTGGAATGCCAATCACTCT and CTGAGCCATCAAAGCCAG for CR3, (T7)CTGGCTTTGATGGCTCAG and CTGCCATTGATAGCAGGATT for CR4, (T7)AATCCTGCTATCAATGGCAG and TCACAGTTTCCGCTCCACAG for CR5, (T7)TCTGCCCTAGCGCTCTCTTCC and GAAATACGGACTTTAATG for 3′UTR, (T7)TCTGCCCTAGCGCTCTCTTCC and CTACTCCCAGCTCTGACAC for 3′UTR1, (T7)TTGTGTCAGAGCTGGGAGTAGand TTGTTCCCCCTTGCCCAG for 3′UTR2, (T7)GTCACCCTTCTGGGCAAGG and CAAGAAGGTGTTGGCATCCC for 3′UTR3, (T7)GACAGGGATGCCAACACCTT and GTCTCTGGGCGATCCCAGTT for 3′UTR4, and (T7)CTGGGATCGCACCTTTTAT and GAAATACGGACTTTAATG for 3′UTR5. These PCR products were transcribed *in vitro* following the manufacturer's instructions (Thermo Fisher Scientific). The *SHC* 5′UTR, CR, and 3′UTR mutants [5′UTRm (bearing C62G, C63G, C81G, C89G, and C100G), CRm (bearing C986G), and 3′UTRm (bearing C2052G, C2379G, C3121G, C3122G, and C3184G)] were prepared by overlapping PCR. The *p16* 3′UTR and CR fragments were described previously [22].

### Bisulfate RNA sequencing

This method could identify the methylation site (m5C) within a RNA fragment shorter than 150 nt. Briefly, fragments 5′UTR1 (positions 1-121), CR3a (positions 868-1013), CR3b (positions 977-1106), CR3c (positions 1062-1199), 3′UTR1 (positions 1928-2066), 3′UTR3 (positions 2296-2446), and 3′UTR5 (positions 3058-3200), which were methylated by NSUN2, were amplified by using following primer pairs: (T7)AGAGAAGAGGCTCCACGTTG and TCTTAACCTTCCTACCATTCC CCTCTGTGGCCCAGGAGTCAC for 5′UTR1, (T7)TGAAATTTGCTGGAATGCC and CCTTCCTACCATTCCATACTCGGCTGTGTCCGGAT for CR3a, (T7)ATTTGCATCCGGCGGGGATC and CCTTCCTACCATTCCAATGGTGCTGATGACATCCT for CR3b, (T7)TGGAGTGTCCCGAAGGGCTT and AACCTTCCTACCATTCCTAGCCATCAAAGCCAGCC for CR3c, (T7)TGTGGAGCGGAAACTGTGAT and ACCTTCCTACCATTCCCTACTCCCAGCTCTGACAC for 3′UTR1, (T7)AAGGGGGAACAAATCACACC and TCTTAACCTTCCTACCATTCC ATTGAAGAATTCAGGGGTCC for 3′UTR2, and (T7)TTTTACAGCTCTTGGCATTT and ACCTTCCTACCATTCCTGCCTCTCAGTCTCGCGGT for 3′UTR5. These fragments (1 μg each) were transcribed *in vitro* and methylated by using non-isotopic SAM. Samples then were dissolved in 10 μl of RNase-free water and mixed with 42.5 μl 5 M sodium bisulfate (Epitect) and 17.5 μl DNA protection buffer (Epitect), incubated in 70°C for 5 minutes and 60°C for 60 min, repeating for 3-5 cycles. After desalting using Micro Bio-spin6 columns, samples were de-sulfonated by 1 M Tris (pH 9.0, 1/1, V/V) at 37°C for 1 h, followed by ethanol precipitation. The bisulfate-converted fragments (0.2 μg) were reverse-transcribed by RevertAid First Strand cDNA Synthesis Kit (Thermo) using primer CGAGGTATTCGCACTGGATACGACTCTTAACCTTC CTACCATTCC. The reverse-transcribed products (cDNA) were amplified by PCR by using following primer pairs: GGAGAGTTTGAGAGAAGAGG and GCAGGGTCCGAGGTATTC for 5′UTR1, GGAGAGTTTGTTGAAATTTG and GCAGGGTCCGAGGTATTC for CR3a, GGAGA GTTTGTTGGAATTTG and GCAGGGTCCGAGGTATTC for CR3b, GGGAGAGTTTGTTTGGAGTGT and GCAGGGTCCGAGGTATTC for CR3c, GGAGAGTTTGTTGTGTGGAG and GCAGGGTCCGAGGTATTC for 3′UTR1, GGAGAGTTTGTAAGGGGGAA and GCAGGGTCCGAGGTATTC for 3′UTR3, and GGAGAGTTTGTTGGGTTTA and GCAGGGTCCGAGGTATTC for 3′UTR5. The PCR products were inserted into the pGEM-T Easy Vector System (Promega). The plasmids purified from single clones were sequenced, the sequences were aligned with the corresponding *SHC* mRNA sequence and the cytosines retained were considered to be methylated.

### *In vitro* translation assays

For *in vitro* translation assays, a cell-free translation system (Promega) in rabbit reticulocyte lysate (RL) was used. Luc-5′UTR, Luc-5′UTRm, Luc-CR, Luc-CRm, Luc-3′UTR, and Luc-3′UTRm fragments were amplified by PCR by using primer pairs (T7)ATGGGGCCTGAAACTGTCTG and ATTACACGGCGATCTTTCCG (for Luc-5′UTR and Luc-5′UTRm), (T7)ATGGAAGACGCCAAAAACAT and TCACAGTTTCCGCTCCACAG (for Luc-CR and Luc-CRm), and (T7)ATGGAAGACGCCAAAAACAT and GAAATACGGACTTTAATGAAG (Luc-3′UTR and Luc-3′UTRm). The Luc-5′UTR, Luc-5′UTRm, Luc-CR, Luc-CRm, Luc-3′UTR, and Luc-3′UTRm fragments then were *in vitro* transcribed and further methylated by NSUN2 *in vitro* or kept untreated. The methylated and non-methylated transcripts (0.01 nM) were used for *in vitro* translation assays. The translation efficiency was determined by measuring the activity of firefly luciferase.

### LC-MS analysis

*In vitro* methylated RNA fragments (1 μg) were digested by nuclease P1 (Sigma) and alkaline phosphatase (Thermo Fisher Scientific). The formation of m5C or m6A was analyzed by HPLC-MS analysis at Tsinghua University Mass Spectrum Center (Beijing, China).

### Measurement of methylation in cells

For methylation assays in cells, 1 μg of anti-m5C antibody (Abcam, Cambridge, MA), 20 μg of cellular RNA, and 20 μl (in 50% slurry) protein-G Sepharose were incubated in IPP buffer (150 nmol/L NaCl, 0.1% NP-40, 10 mmol/L Tris-HCl [pH 7.4]) plus 1 U/μl RNasin in 500 μl at 4°C for 2 h. The IP beads then were washed 5 times with IPP buffer. RNA isolated from the IP beads was subjected to real-time qPCR analysis.

### Western blot analysis

Western blot analysis was performed as described [6]. Monoclonal anti-GAPDH, polyclonal anti-p66SHC was from BD Biosciences. Monoclonal anti-p38, monoclonal anti-TP53, polyclonal anti-p-p38, and polyclonal anti-p16 were from Santa Cruz. Polyclonal anti-cyclin A and polyclonal anti-cyclin B1 were from Abcam. After secondary antibody incubations, signals were detected by SuperSignal WestPico Chemiluminescent Substrate (Pierce) following the manufacturer's instruction and quantitated by densitometric analysis with ImageMaster VDS software.

## SUPPLEMENTARY MATERIAL FIGURES


