# Supplementary material for: RNA methyltransferase NSUN2 promotes stress-induced HUVEC senescence
Source: Oncotarget. 2016 Mar 15;7(15):19099–110. doi: 10.18632/oncotarget.8087 (PMC4991368; doi:10.18632/oncotarget.8087)
Supplement: Supplementary file 1 [file oncotarget-07-19099-s001.pdf]

RNA methyltransferase NSUN2 promotes stress-induced HUVEC senescence

Supplementary Material

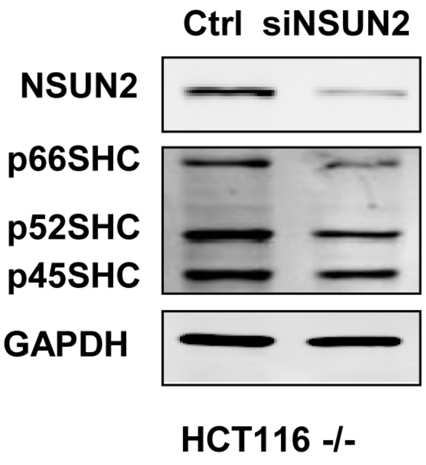

Supplemental Figure S1. NSUN2 regulates SHC expression in a TP53-independent manner. TP53-deficient HCT116-/- cells were transfected with a siRNA targeting NSUN2. Forty-eight hours later, cell lysates were prepared and subjected to Western blot analysis to assess the levels of proteins NSUN2, p66SHC, p52SHC, p46SHC, and GAPDH.

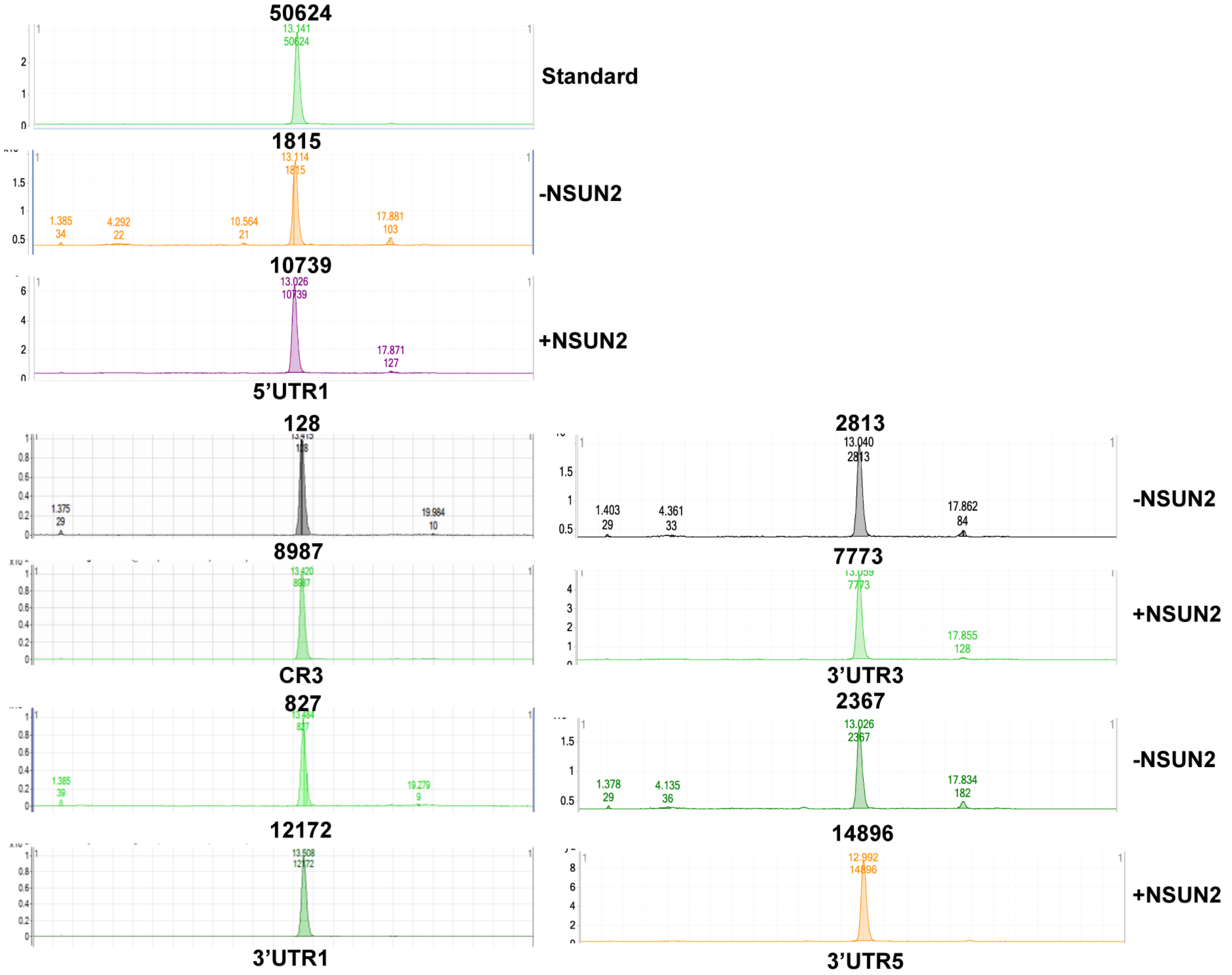

Supplemental Figure S2. Measurement of m5C in NSUN2-methylated *SHC* mRNA fragments. *In vitro* methylated (+NSUN2) or unmethylated (-NSUN2) *5’UTR1, CR3, 3’UTR1, 3’UTR3, and 3’UTR5* fragments were subjected to HPLC-MS analysis to determine the formation of m5C. The peak value of m5C is indicated at the top of the peaks.

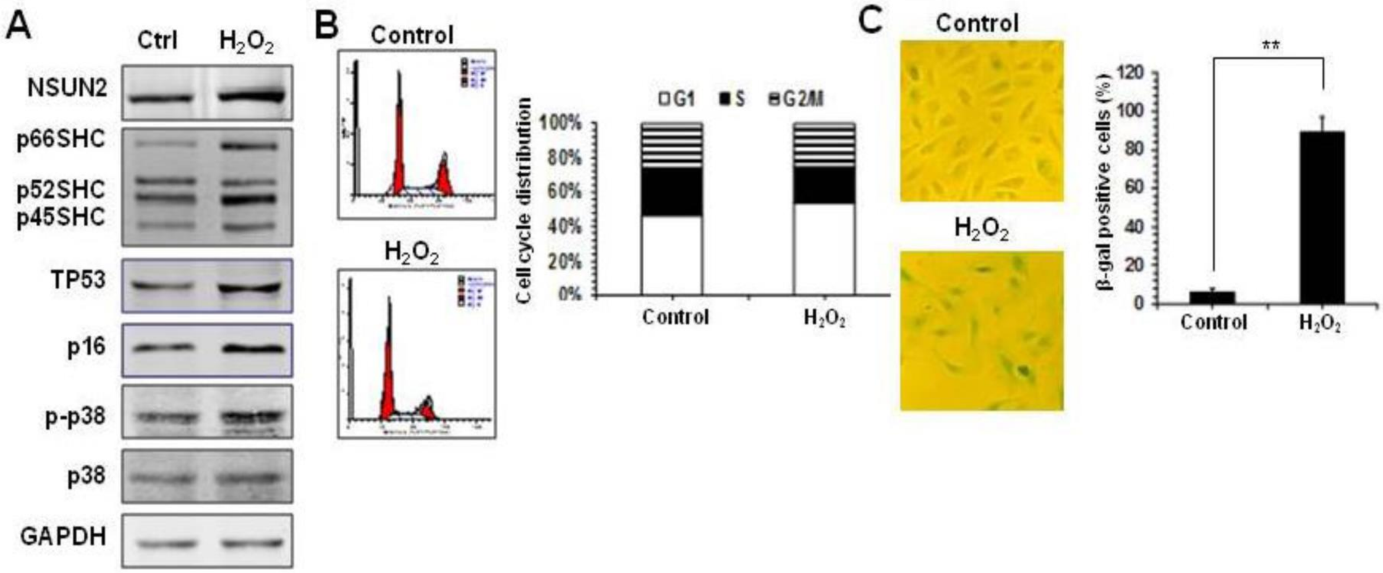

Supplemental Figure S3. Elevation of NSUN2 and SHC proteins in oxidative stress-induced cellular senescence. **(A)** HUVEC cells were exposed to H_2O_2 (30 μM). Seventy-two hours later, cell lysates were prepared and subjected to Western blot analysis to assess the levels of proteins NSUN2, p66SHC, p52SHC, p46SHC, TP53 (p53), p16, p-p38, p38, and GAPDH. **(B)** Cells described in Fig. S3A were used for FACS analysis. Data are representative from 3 independent experiments. **(C)** Cells described in Fig. S3A were subjected to SA-β-gal staining. Data represent the mean±SD from 3 independent experiments; significance was analyzed by Students' *t* test (**, $p<0.01$).

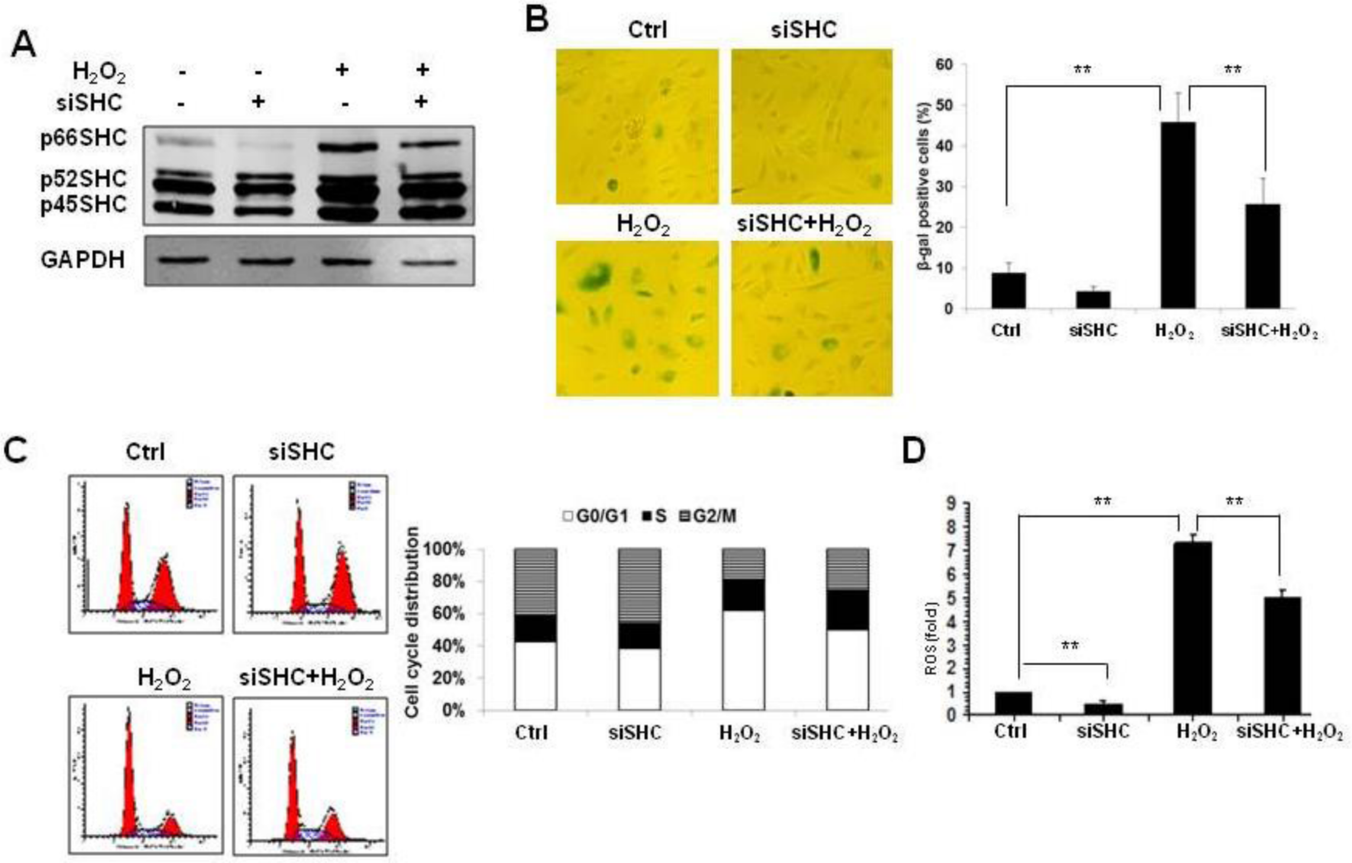

Supplemental Figure S4. SHC knockdown diminishes the effect of oxidative stress in inducing cellular senescence. **(A)** HUVECs were transfected with an SHC-directed siRNA or a control siRNA. Twenty-four hours later, cells were exposed to H_2O_2 (30 μM) and cultured for an additional 48 h. Cell lysates were prepared and subjected to Western blot analysis to assess the levels of p66SHC, p52SHC, p46SHC, and GAPDH. **(B)** Cells described in Fig. S4A were subjected to SA-β-gal analysis. Data represent the means ± SD from 3 independent experiments and statistical significance was analyzed by Student's *t* test (**, $p<0.01$). **(C)** Cells described in Fig. S4A were subjected to FACS analysis. Data are representative from 3 independent experiments. **(D)** Cellular ROS levels in cells described in Fig. S4A were analyzed. Data shown are the means ± SD from 3 independent experiments and statistical significance was analyzed by Student's *t* test (**, $p<0.01$).

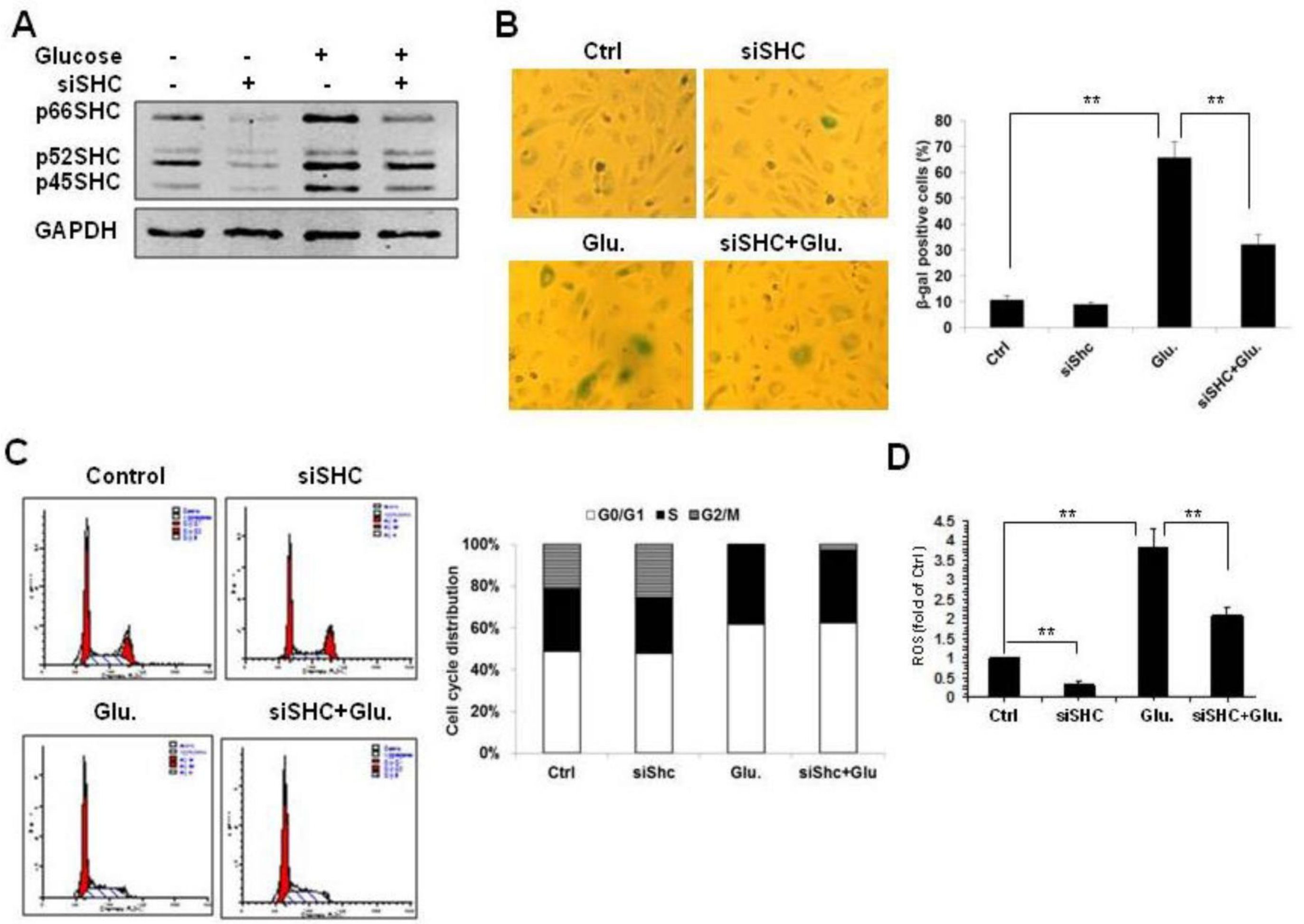

Supplemental Figure S5. SHC knockdown diminishes the effect of high glucose in inducing cellular senescence. **(A)** HUVECs were transfected with an SHC siRNA or a control siRNA. Twenty-four hours later, cells were exposed to high glucose (33 mM) and cultured for an additional 48 h. Cell lysates were prepared and subjected to Western blot analysis to assess the levels of p66SHC, p52SHC, p46SHC, and GAPDH. **(B)** Cells described in Fig. S5A were subjected to SA-β-gal staining. Data represent the means ± SD from 3 independent experiments and statistical significance was analyzed by Student's *t* test (**, $p<0.01$). **(C)** Cells described in Fig. S5A were subjected to FACS analysis. Data are representative from 3 independent experiments. **(D)** Cellular ROS levels in cells described in Fig. S5A were analyzed. Data shown are the means ± SD from 3 independent experiments and statistical significance was analyzed by Student's *t* test (**, $p<0.01$).

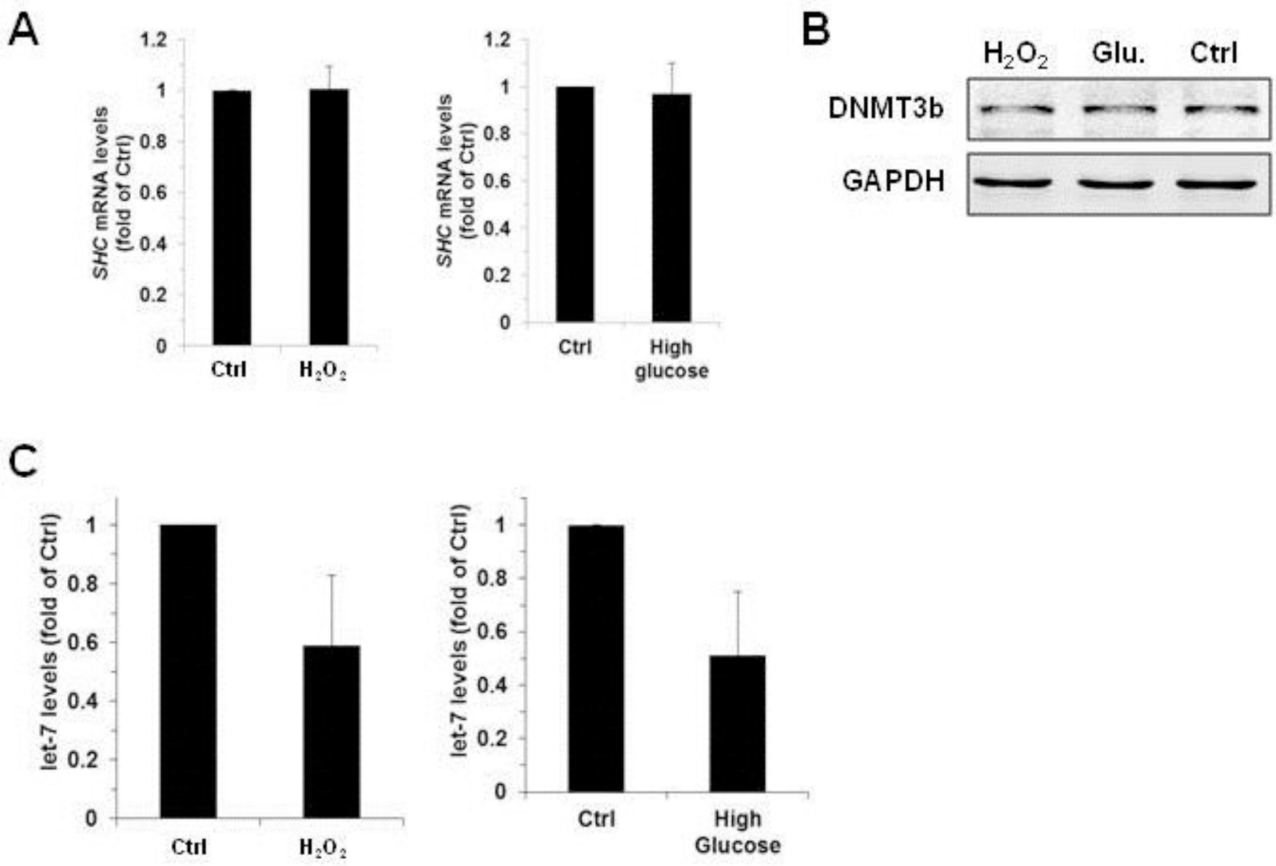

Supplemental Figure S6. Influence of H_2O_2 and high glucose on DNMT3b and let-7 expression levels. **(A)** HUVECs were exposed to H_2O_2 (30 µM) or high glucose (33 mM). Seventy-two hours later, RNA was prepared and subjected to RT-qPCR analysis to assess the levels of *SHC* mRNA. Data represent the means ± SD from 3 independent experiments. **(B)** HUVECs were exposed to H_2O_2 (30 µM) or high glucose (33 mM) and 72 h later, cell lysates were prepared and subjected to Western blot analysis to assess the levels of DNMT3 and GAPDH. **(C)** RNA prepared from cells described in Fig. S6A was subjected to RT-qPCR analysis to assess the levels of let-7. Data represent the means ± SD from 3 independent experiments (*, $p<0.05$).
